# A Rare T2-T3 Synovial Facet Cyst Causing Progressive Myelopathy

**DOI:** 10.1155/2021/2799749

**Published:** 2021-06-29

**Authors:** Mohamed F. Albana, Sean Z. Griffiths, Kris E. Radcliff

**Affiliations:** ^1^Department of Orthopedic Surgery, Inspira Heath, 1505 W Sherman Ave, Vineland, NJ 08360, USA; ^2^Department of Orthopedic Surgery, The Rothman Institute, 2500 English Creek Ave. Egg Harbor Township, NJ 08234, USA

## Abstract

Intraspinal extradural synovial cysts are a rare occurrence at the spinal cord level and thus a rare cause of myelopathy. Synovial cysts usually present in the more mobile lumbar and cervical parts of the spine; however, they may also arise in the thoracic spine. We present a case of a 59-year-old male with a left upper thoracic synovial cyst at T2-3 causing disabling, progressive myelopathy, and an incomplete spinal cord injury syndrome with inability to ambulate. An urgent decompressive laminectomy with bilateral facetectomies, cyst excision, and posterior fusion was performed. Subsequently, the patient recovered full function. Synovial cysts should be considered in the differential diagnosis of progressive thoracic myelopathy. This is only the sixth reported case of a synovial cyst of this kind occurring between the levels of T1 and T7. Urgent surgical decompression is the recommended treatment.

## 1. Introduction

Intraspinal extradural synovial cysts are a rare cause of myelopathy, and when they occur, they most commonly present in the lumbar and cervical spine. Such cysts can also appear in the thoracic spine, although such cases are rare. We present a case of a 59-year-old male with a left-sided intraspinal, extradural synovial cyst at the T2-T3 levels on the left resulting in an inability to ambulate and paresthesia in bilateral lower extremities. A laminectomy and excision of the cyst with fusion from T2-T4 was performed resulting in complete resolution of symptoms. There are only 5 reported cases of synovial cysts in the upper thoracic spine. Laminectomy, with cyst excision, is the treatment of choice, although steroid injections and percutaneous drainage have been described in the literature.

## 2. Case Report

### 2.1. History

Patient is a 59-year-old male who sustained a minor impact to his back one month prior to presentation. He was evaluated by a pain management specialist for chief complaint of back pain and ambulatory dysfunction. An MRI of the lumbar spine was performed which demonstrated mild, multilevel spondylosis. He was referred for physical therapy of the lumbar spine and was prescribed NSAIDs and muscle relaxers. He was reevaluated and reported worsening gait and an improvement in pain. He was referred for a lumbar epidural injection. He was reevaluated again two weeks after the epidural (approximately six weeks following the initial injury). At that time, he was completely wheelchair bound. He endorsed difficulty with bladder control for two days. He was referred to the emergency department for an immediate evaluation. At that time, he reported mild (3/10) pain in the lumbar spine. He denied neck pain, thoracic pain, arm pain, or leg pain. He denied numbness or paresthesia in any distribution including the chest wall.

### 2.2. Examination

The patient's physical examination revealed 2/5 strength in the left iliopsoas, quadriceps, tibialis anterior, EHL, and gastrocnemius. His right lower extremity strength was 4/5 in the left iliopsoas, quadriceps, tibialis anterior, EHL, and gastrocnemius. He had decreased sensation below the nipple line bilaterally. He had normal digital rectal tone. He had no sensory loss in the perineal region. He had no clonus or Babinski sign. He had 1+ reflexes in all distributions.

### 2.3. Imaging

**Figure 1:**
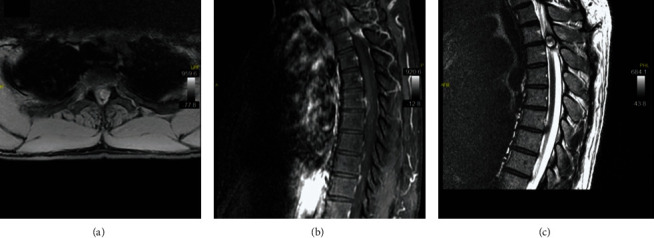
(a) Axial T1-weighted MRI, (b) sagittal T1-weighted MRI, and (c) sagittal T2-weighted and short tau inverted recover MRI images demonstrating a large left T2-T3 synovial facet cyst within the spinal canal.

A CT scan of the thoracic spine was ordered and found to be negative for any acute pathologic process. An MRI of the thoracic spine demonstrated a large high T2 intensity, low T1 intensity, heterogenous, and extradural round mass at the level of T2-T3 on the left side. There was no contrast enhancement. There was severe cord compression. There was acute cord signal change at the T2 level (Figure 1).

The patient was admitted to the intensive care unit, and intravenous steroids were administered with no significant improvement prompting surgical management.

### 2.4. Surgical Intervention

**Figure 2:**
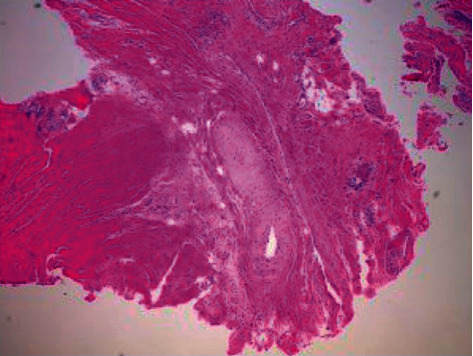
Microscopic image showing denuded cyst lining with focal synovium present. Gross specimen size 4.0 × 1.3 × 0.5 cm.

The patient was taken to the operating room for surgical intervention. Due to the location of cyst, as noted on preoperative imaging, a full laminectomy of T3 was performed first to define a normal region of dura. During the approach, the T3-4 interspinous ligament was resected. A midline laminectomy and decompression of T1, T2, and T3 was performed followed by complete excision of a large 3 cm × 1.5 cm synovial cyst (Figure 2). The cyst was noticed to be originating from the left T2-T3 facet and demonstrated adhesions to the dura but was clearly extradural. Medial facetectomies and foraminotomies from T2 to T4 were performed bilateral to help prevent cyst recurrence and ensure a wide decompression followed by instrumented spinal fusion from T2 to T4 with autograft and allograft. The fourth thoracic vertebra was included in the fusion to minimize risk of iatrogenic instability as the T3-4 interspinous ligament was resected during the approach. There were no intraoperative complications or adverse recorded signal changes on intraoperative monitoring from baseline.

### 2.5. Postoperative Course

**Figure 3:**
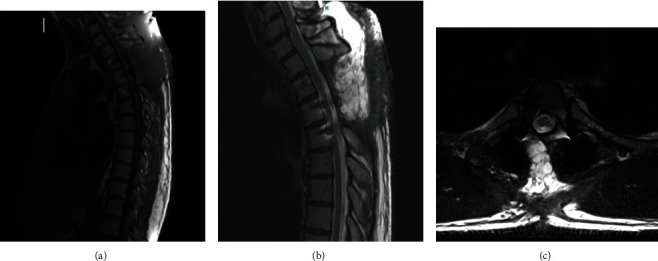
Sagittal, precontrast (a) and postcontrast (b), and axial postcontrast (c) MRI sequences 3 months postoperatively demonstrating maintained surgical decompression and no recurrent cyst formation.

Immediately after surgery, the patient was at his preoperative baseline level. He had an uneventful postoperative course. He was discharged to an inpatient rehabilitation facility and eventually home. Final pathology report resulted in a diagnosis of a benign synovial cyst. The patient was evaluated at 2, 6, and 10 weeks postoperatively. At his two-week follow-up, his strength had substantially improved to at least four out of five in all distributions. By the sixth week postoperatively, patient's strength was fully restored to five out of five strength bilaterally in all distributions with no remaining paresthesia. At the patients tenth week postoperatively, his strength had remained full with no recurrence of symptoms and complete resolution of symptoms. A follow-up MRI was obtained three months postoperatively and revealed persistently adequate decompression of the spinal cord with no cyst recurrence as compared to preoperatively (Figure 3).

## 3. Discussion

We present a case of a relatively young male in the setting of a thoracic synovial cyst. Synovial cysts in themselves are rare in the spine; however, when they are identified, they typically arise in the lumbar spine [1–4]. When they do present in the cervical or thoracic spine, they often arise in locations of increased motion such as the cervicothoracic junction or lower thoracic vertebra [5, 6]. There have been 6 reported cases of extradural synovial cysts appearing in the cervicothoracic junction and 20 cases occurring between T10 and T12 [7, 8]. Other risk factors for the formation of juxtafacet synovial cysts include rheumatoid arthritis, osteoarthritis, and posttraumatic instability [3]. Giovannini et al. identified 43 cases of thoracic juxtafacet synovial cysts. Only 5 of the 43 cases occurred between T1 and T7, which can be attributed to the anatomical characteristics of the thoracic spine leading to decreased mobility [3, 8, 9].

Patients are typically treated conservatively initially with analgesics, immobilization, and occasionally CT-guided aspiration with, or without, steroid injection [8, 10]. Graham et al. determined the use of CT-guided aspiration as a good option for temporary symptomatic relief. On the other hand, Shah et al. evaluated 10 patients with radiculopathy that were initially treated conservatively and reported that nonsurgical management is not successful and surgery should be recommended for a zero rate of recurrence [10, 11]. In the event a patient develops ambulatory dysfunction, urgent surgical decompression should be completed once an etiology for the dysfunction is identified [12]. Multiple surgical options have been studied and found to be acceptable with laminectomy with, or without, fusion being most commonly used [3, 10, 13–17]. In this case, we opted to perform facetectomies and fusion in addition to decompressive laminectomy to eliminate the pathologic zygapophyseal joints and the associated motion in order to prevent recurrence.

## 4. Conclusion

Juxtafacet synovial cysts are rare, but the rate of diagnosis has increased due to the use of MRI. This pathology is exceedingly rare in the thoracic spine, and upper thoracic presentation is even more infrequent. In patients who develop ambulatory dysfunction, a thorough workup for thoracic pathology should be conducted even in the absence of thoracic pain. The presence of a synovial cyst should prompt continued evaluation as the cyst can increase in size leading to progressive myelopathy. Synovial cysts do not necessarily illicit axial back pain. In the case presented, it is hypothesized that the minor trauma the patient experienced caused inflammation that may have lead to the synovial cyst expansion. Nonsurgical management has been described; however, surgical excision is recommended as symptoms commonly persist with nonoperative management. This patient had an incomplete Brown-Sequard presentation, which typically have a good clinical prognosis. Facetectomy with posterior spinal fusion after cyst excision should be determined on a case by case basis but should be considered to help prevent cyst recurrence. When ambulatory dysfunction is present, operative intervention should be urgently completed to optimize patients' chance of improvement.
